# Clinical Significance of Tie-2-Expressing Monocytes/Macrophages and Angiopoietins in the Progression of Ovarian Cancer—State-of-the-Art

**DOI:** 10.3390/cells11233851

**Published:** 2022-11-30

**Authors:** Wiktoria Skiba, Dorota Suszczyk, Anna Pawłowska, Karolina Włodarczyk, Anna Pańczyszyn, Iwona Wertel

**Affiliations:** 1 Independent Laboratory of Cancer Diagnostics and Immunology, Department of Oncological Gynaecology and Gynaecology, Faculty of Medicine, Medical University of Lublin, Chodźki 4a, 20-093 Lublin, Poland; 2 Institute of Medical Sciences, Department of Biology and Genetics, Faculty of Medicine, University of Opole, Oleska 48, 45-052 Opole, Poland

**Keywords:** ovarian cancer, tumour microenvironment, Tie-2-expressing monocytes, angiopoietins, angiogenesis

## Abstract

Tumour growth and metastasis are specific to advanced stages of epithelial ovarian cancer (EOC). Tumour angiogenesis is an essential part of these processes. It is responsible for providing tumours with nutrients, metabolites, and cytokines and facilitates tumour and immune cell relocation. Destabilised vasculature, a distinctive feature of tumours, is also responsible for compromising drug delivery into the bulk. Angiogenesis is a complex process that largely depends on how the tumour microenvironment (TME) is composed and how a specific organ is formed. There are contrary reports on whether Tie-2-expressing monocytes/macrophages (TEMs) reported as the proangiogenic population of monocytes have any impact on tumour development. The aim of this paper is to summarise knowledge about ovarian-cancer-specific angiogenesis and the unique role of Tie-2-expressing monocytes/macrophages in this process. The significance of this cell subpopulation for the pathophysiology of EOC remains to be investigated.

## 1. Introduction

Ovarian cancer (OC) is a gynaecological cancer characterised by a high mortality rate. This asymptomatic cancer was diagnosed in 313,959 women worldwide in 2020, resulting in 207,252 deaths in the same year [1]. The most-frequent histological subtype of OC is epithelial ovarian cancer (EOC), accounting for 90% of OC cases. The existing knowledge about and updates on OC are summarised in De Leo et al., highlighting the World Health Organisation (WHO) classification of OC, especially the enrichment of modern diagnostic criteria with new histopathological, immunohistochemical, and molecular data [2,3]. The prognosis of EOC is unfavourable. Most patients are in advanced stages—III or IV according to the International Federation of Gynaecology and Obstetrics (FIGO) classification, usually at stage IIIC when diagnosed [4]. The cancer starts to spread at stage II in the pelvic region, and then, at stage III, it metastases within the abdomen. At stage IV, cancer cells spread outside the abdomen to the distant organs [5]. Angiogenesis is a significant contributor to the progression of OC. Blood vessels form in the tumour as a result of a variety of neovascularisation mechanisms, such as sprouting angiogenesis, intussusceptive angiogenesis, vasculogenesis, recruitment of endothelial progenitor cells, vascular mimicry, and transdifferentiation of cancer stem cells [6]. Angiogenesis enables the mobility of neoplastic cells, as well as immune cells within the tumour, thus allowing metastasis [7,8] and regulating immune cells’ inflow into the tumour, along with their activation [9]. 

Among the immune cells, there is a specific subpopulation of monocytes—Tie-2-expressing monocytes/macrophages (TEMs). The role of TEMs in EOC patients remains unclear, and the data on their functions are limited. The significance of this cell subpopulation in the regulation of ovarian tumour growth, angiogenesis, and metastasis of EOC remains to be investigated.

The aim of this study is to summarise knowledge about OC-specific angiogenesis and to help understand the unique role of TEMs in this process.

## 2. Vasculature of the Ovaries in Health and Disease

Angiogenesis is a normal physiological mechanism defined as the formation of new blood vessels from already existing ones [6,10]. The modulation of this multistep process is highly orchestrated by (1) antiangiogenic factors, i.e., angiopoietin (Ang), thrombospondin 1 (TSP-1), and endostatin, and (2) proangiogenic factors, which include the vascular endothelial growth factor (VEGF) family, i.e., VEGF A-E, the placental growth factor (PLGF), fibroblast growth factor 2 (FGF-2), matrix metalloproteinase 7 (MMP-7), the platelet-derived growth factor (PDGF), tumour-derived growth factors (TGF-α and TGF-β), tumour necrosis factor α (TNF-α), prostaglandin E2 (PGE2) [11], and interleukin 8 (IL-8), also called CXCL-8, with its receptor CXCR2 [12]. When the balance between these two groups is disturbed in favour of proangiogenic factors, an “angiogenic switch” occurs, triggering blood vessel formation [6].

Except for neoplasia and some non-cancerous processes—such as wound healing—which require blood vessel development, the vascular system in adults is in a quiescent state. Other exceptions are the female reproductive organs, especially the endometrium and ovaries. The ovary goes through cyclic changes, and its physiology depends on the periodic formation, differentiation, and regression of ovarian vasculature. Normally, the ovary exhibits increased permeability of the blood vessels during follicular development, ovulation, and the subsequent formation of the corpus luteum [13,14]. These processes are disrupted in the ovaries during bulk development [14]. Another process that is disturbed and deregulated by cancer is angiogenesis [15]. When the tumour manages to hijack this process, the number of tumour blood vessels grows (increased microvessel density), and these vessels have an abnormal structure (increased permeability) compared to the normal ones [9]. Dysfunctional vascular morphology—and, by extension, vessel malfunction—is the result of a poorly organised structure of tumour vasculature. This is caused by the loss of pericyte–endothelial cell interactions and their endothelial junctions with each other, as well as by basement membrane impairment [16]. Angiogenesis is a process of great significance in tumour development, since it is responsible not only for tumour formation and metastasis, but also for chemoresistance and relapse. Critical to these processes are also auspicious revascularisation and restoration of perfusion [17].

## 3. Components of the EOC Environment

The integrity of the abdominal cavity serves to provide intra-abdominal organs with inflammatory cell populations during pathological events. However, it also creates a conductive environment for cancer progression [18]. Malignant ascites acts as a transporter, facilitating the spread of highly carcinogenic tumour cells to the pelvic and peritoneal cavities [19]. At onset, the cancer is limited to the ovaries. As the disease progresses, intra-abdominal organs and the peritoneum become invaded [5]. Originally restricted to a microenvironment, the process thus extends to a more complex and much larger environment. The TME plays a significant role in EOC progression. Multiple studies described in Yang et al. [11] indicated that it is a highly complex, dynamic, cross-talking niche with various potential therapeutic targets.

The TME is composed of two general parts—(1) the extracellular matrix (ECM) and (2) stromal cells [11]. One of its distinctive features is hypoxia. According to literature data, hypoxia is involved in processes such as tumour growth, angiogenesis, resistance to apoptosis, and chemotherapy, as well as metastasis [20]. The ECM includes chemokines, inflammatory cytokines, integrins, and MMPs. The latter group, stromal cells, comprise cancer-associated fibroblasts, endothelial cells, pericytes, tumour cells, cancer stem cells, and immune cells [11].

Immune cells present in the TME may function as a double-edged sword based on their role in the pro- or antitumoural response. The immune cells acting as tumour enemies are cytotoxic T lymphocytes (CD8^+^) and activated helper T-cells. The allies of the tumour are myeloid-derived suppressor cells (MDSCs), tumour-associated macrophages (TAMs), lymphocyte T helper cells (Th2 subtype), and T regulatory cells (Tregs) [19]. Monocytes can play a dual role [21]. On the one hand, they exhibit strong anti-cancer effects. On the other hand, these cells can show pro-tumour activity [22]. It is worth mentioning that monocytes impact other immune cells present in the TME. Monocytes interact with the adaptive immune system by directing the recruitment and function of lymphocytes within the TME through paracrine signalling. They also function as antigen-presenting cells [22]. Several research teams showed that monocytes closely interact with natural killer (NK) cells, being involved especially in the recruitment and activation of NK cells within the TME [21,23,24,25,26]. The ratio between monocytes and NK and T-cells is a critical factor in the TME. If there are more monocytes than functionally competent NK and T-cells, monocytes show protumoural activity. Monocytes’ interaction with tumour cells may enhance tumour cells’ resistance to death initiated by NK or T-cells. Kaur et al. [27] showed the elevation of monocytes and a decrease in NK or CD8^+^ T-cells in terminally ill pancreatic cancer patients at the later stages of cancer progression. On the contrary, if there are fewer monocytes than competent NK and T-cells, it is more likely that they interact with each other. Therefore, it is likely that they will increase the functional capabilities of NK and T-cells to target the tumour cells [21]. In addition, NK cells can eliminate monocytes, which prevents them from interacting with tumours directly within the tumour microenvironment [28]. Studies by Gordon and Freedman [29] showed that monocytes isolated from the peripheral blood (PB) or peritoneal fluid (PF) of ovarian cancer patients exhibited a decreased capacity for antibody-dependent cytolysis and phagocytosis of tumour cells upon in vitro activation [19].

In EOC patients, monocytes/macrophages infiltrate the tumour tissue. Furthermore, their ability to stimulate angiogenesis and excrete immunosuppressive factors seems to have an impact on EOC progression [30,31]. According to Zhang et al.’s [32] meta-analysis, macrophage infiltration of ovarian cancer correlates with a worse prognosis. However, another study has shown that TAMs’ infiltration is closely linked to a good prognosis in ovarian cancer patients [33,34]. 

Studies by Wang et al. [18,35] showed that there are notable differences between the peritoneum of patients with benign pelvic tumours and EOC patients. These differences primarily relate to the structure and inflammatory status. Structural differences include thickening or oedema, enhanced vascular patterns, and soft or firm adhesions. However, certain similarities to peritonitis have been found as well, including the florid appearance of the peritoneum and intestinal serosa [36]. The differences in the inflammatory status of the tissue and PF are mostly observed in the monocyte/macrophage phenotype among EOC patients and those with benign conditions. More than 75% of the immune cells found in the peritoneum of EOC patients were CD68 positive and showed an M2 phenotype [18]. According to the authors, this significant infiltration by macrophages mimics the chronic inflammation of the peritoneum in EOC patients [18,35].

Since OC is a heterogeneous disease with a highly complex TME, innovative and more sophisticated methods of studying this cancer are needed. Currently, the most common methods are 2D and 3D cell cultures and xenografts [37]. According to the latest reports, the most efficient way to study EOC would be organoids. This model is a patient-derived 3D culture (PD3D) that faithfully recapitulates heterogeneity and the histological and genomic features of primary tumours [38,39]. There are reports on the possibility of xenografting OC organoids [38]. This would be a highly valuable addition to the already considerable advantages of xenografts compared to the other models. This is due to their ability to imitate the interplay between tumour cells and TME components, such as blood vessels [37].

The literature indicates that one of the subsets of monocytes/macrophages—Tie-2-expressing monocytes/macrophages—plays a key role in the TME, tumour progression, and tumour spread [40,41].

## 4. Tie-2-Expressing Monocytes

### 4.1. Distinguishing Feature—Tie-2 Receptor

Tie-2-expressing monocytes/macrophages are a recently discovered subset of monocytes [35]. First described by De Palma et al. [42], these myeloid cells are characterised by some idiosyncratic features [43]. First and foremost, they express Tie-2 (receptor tyrosine kinase with Ig and EGF homology domains, TEK, or CD202b), which was, together with Tie-1, originally identified as an orphan receptor. Currently, it is defined as a part of the second endothelial-cell-specific receptor Tyr kinase signalling system [44]. TEM presence and specific phenotype—CD45^+^ Tie-2^+^—have been indicated to be present in human blood [30]. The largest number of them have been detected in the perivascular space. The TME formed after cancer treatment with irradiation and chemotherapy is found in the area where TEMs accumulate. Their role is to invigorate temporary localised vascular permeability and promote tumour cell intravasation [17,45,46]. TEMs are assumed to be proangiogenic since, compared to Tie-2^−^ monocytes, they have upregulated *MMP-9, VEGFA, COX-2,* and *WNT5A* genes [47]. The phenotype of TEMs is slightly different in humans and a murine model—CD45^+^Tie-2^+^CD11b^+^Gr-1^low/neg^ from the latter group and CD45^+^Tie-2^+^CD11b^+^CD11c^+^CD14^low^CD16^+^ in humans. Tie-2^+^Sca-1^+^CD11b^+^ markers distinguish TEMs from other monocytes in PB [30]. Despite phenotype differences between TEMs and other monocytes, gene expression analysis suggests that TEMs, embryonic and foetal macrophages, resident blood monocytes, and tumour-infiltrating TEMs express genes characteristic of TEMs. This suggests that these myeloid populations represent the developmental stages of TEMs [48]. It has been shown that TEMs are the major subset of mononuclear cells expressing Tie-2 on their surface during PB examination [35,49].

TEMs accounted for most of the angiogenic activity of bone-marrow-derived cells. Murine tumour models showed that TEMs’ knockout completely averted tumour neovascularisation [50]. As a consequence, their selective elimination attenuated angiogenesis and tumour growth [49]. However, another study on a murine model with *Tie-2* deletion in myeloid-derived cells did not confirm its impact on metastasis [17]. 

Other studies have indicated that the deficiency of TEMs restricts the “angiogenic switch” [11,31]. Some reports suggest their role in tumour metastasis due to their ability to secrete VEGF-A, resulting in increased vascular permeability [45,51]. 

It should be emphasised that there are only two studies indicating TEMs’ presence in EOC [35,52] and several more on the Tie-2 receptor and its ligand—Ang-2—in EOC patients and murine EOC models [8,35,53,54,55,56,57,58,59,60,61,62,63,64,65]. Data on human studies concerning Tie-2/Ang-2 expression in EOC patients are presented in Table 1.

### 4.2. The Role of Tie-2-Expressing Monocytes in EOC Progression and Metastasis

It should be stressed that the available data on the role of TEMs in EOC progression and metastasis are scarce. Conejo-Garcia et al. [66] identified vascular leukocytes (CD14^+^CD45^+^CD11c^+^CD11b^+^VE-Cadherin^+^CD31^+^CD146^+^) in the tumour tissue of EOC patients. Their later study indicated that the majority of these cells also expressed the Tie-2 receptor [52]. Wang et al. [35] showed the increased presence of TEMs, identified as CD14^+^Tie-2^+^ cells, in the tumour tissue, PB, and peritoneal fluid of EOC patients. The number of TEMs in tumour tissue was positively correlated with microvessel density. A murine model study has shown that TEMs are able to promote tumour angiogenesis and metastasis [35].

Angiopoietins 1-4 are ligands for the Tie-2 receptor [67], among which Ang-1, Ang-2, and Ang-4 are detected in humans [16].

Ang-1 seems to have no impact on TEMs. However, Ang-2 drives TEMs’ proangiogenic genes [47] and the expression of Tie-2 on their surface [68,69]. Coffelt et al. [47] showed that tumour-derived Ang-2 caused the upregulation of thymidine phosphorylase and cathepsin B genes, known as proangiogenic enzymes, as well as immunosuppressive ones, such as IL-10, mannose receptor (MRC1), and CCL17. The subsequent investigation of this group indicated that TEMs inhibit the T-cell response and stimulate (Tregs’ differentiation. This effect is presumed to be directly related to the anti-inflammatory activity of IL-10, which they secrete as a result of Ang-2 stimulation. In addition, TEMs are responsible for inhibiting T-cell proliferation and weakening T-cell response, which is crucial in anticancer response [70]. Other reports showed that Ang-2 inhibition caused the downregulation of Tie-2 in TEMs, thus decreasing their association with vasculature and angiogenic potential in the metastatic mammary-specific polyomavirus middle T-antigen overexpression mouse model (MMTV-PyMT) and a transgenic mouse model of β-cell carcinogenesis RIP1-Tag2 (pancreatic insulinomas) [67,68]. What is more, Wang et al. [35] detected an elevated secretion of insulin-like growth factor 1 (IGF1) by TEMs after Ang-2 stimulation. The authors concluded that circulating Ang-2 could upregulate the expression of IGF1 in TEMs. It was previously shown that, in pathological conditions, IGF factors play an important role in tumorigenesis by inducing antiapoptotic activity [35,71]. Contrary to these reports, Jakab et al. [17] did not observe any significant upregulation on mRNA or protein levels after Ang-2 stimulation.

The data presented in Table 1 indicate that the angiopoietin–Tie-2 (Ang-Tie-2) axis is altered in EOC patients. Circulating Ang-2 is elevated in EOC patients compared to healthy women [35,57,58]. Ang-2 is accumulated in greater concentrations in ascites compared to the matched serum samples obtained from the same patients [35]. However, there are no differences in Ang-2 expression between platinum-sensitive and -resistant EOC patients [62]. The expression of Ang-2 and Tie-2 is detected in EOC tissue at the protein [8,56,61], as well as the gene level [65]. The expression of these two factors in EOC tissue is higher compared to normal ovaries. However, tumour-free peritoneal tissue and metastatic lesions show increased expression of Tie-2 [8] and Ang-2 [8,65] compared to primary tumour tissue. Circulating Tie-2 may also be used as a biomarker of bevacizumab treatment efficiency [60,63,64]. Taken together, these data suggest that the Ang-Tie-2 pathway may play an important role in EOC progression and that it is an interesting target for OC therapy.

### 4.3. Tie-2-Expressing Monocytes among Tumour-Associated Monocytes 

Monocytes and their tissue-infiltrating population—macrophages, innate effectors—are known to play a crucial role in tumour development. Monocytes described by Furth and Cohn and macrophages originally discovered by Élie Metchnikoff as phagocytic cells also have the ability to process and present antigens through major histocompatibility complex (MHC) proteins, as well as to produce and secrete multiple cytokines [72,73]. For almost half a century, researchers have been providing data implicating monocytes/macrophages in angiogenesis in each step of this process [74].

Monocytes and macrophages are large populations of monocytic cells that have long been the central focus of many scientists. Efforts to organise the knowledge about these cells to gain a better understanding of them resulted in a classification based on (1) origin (tissue-resident and bone-marrow-derived) [34], (2) polarisation status/activity (M1, M2 and non-polarised monocytes) [75], and (3) phenotype (classical, intermediate, and non-classical) [76,77,78].

Monocytes are widely divided into two main groups—M1 and M2. The second group, according to Mantovani et al. [75], can be divided into three subgroups: M2a (activated by IL-4 or IL-13; express CD206, CD163, and fibronectin), M2b (activated by Toll-like receptors, ligands, and immune complex activation), and M2c (activated by IL-10). Interestingly, the high plasticity of macrophages causes them to act in the tumour environment under the signalling instructions provided by the tumour. Tumour-associated macrophage polarisation status is dependent on cancer cells present in the TME. The ratio from antitumour M1 changes to protumour M2 status [79]. In the initial stages of EOC, TAMs show mostly the M1 phenotype. Due to EOC progression, these cells are affected by the changed TME. This results in their progressive transformation into the extreme phenotype, which is immunosuppressive and promotes tumour development—M2 macrophages [80].

The M1/M2 classification of macrophages is currently considered to be an oversimplified approach [81]. Scientific reports on TEM classification as M1 or M2 monocytes/macrophages are contradictory. According to the literature, TEMs can be classified as a subtype of M2 macrophages [11,47,48,49,82,83,84,85,86] and M1 macrophages, with recent studies showing Tie-2 expression on proinflammatory macrophages [87,88]. Non-polarised Tie-2-expressing macrophages (i.e., macrophages that are negative for the traditional M1, as well as M2 markers) are also mentioned in the literature [47]. All this indicates that Tie-2 expression is not strictly related to one specific functional monocyte phenotype [88].

Another classification divides monocytes into three groups based on differential expression—mainly the clusters of differentiation CD14 and CD16 [76,77,78] and the 6-Sulfo LacNAc (SLAN) molecule—a carbohydrate modification of P-selectin glycoprotein ligand 1 (PSGL-1) recognised by the monoclonal antibody M-DC8 [78,89]. 

Several studies have demonstrated that classical (CD14^++^CD16^−^SLAN^−^), intermediate (CD14^++^CD16^+^SLAN^−^), and non-classical (CD14^+^CD16^++^SLAN^+^) monocytes exhibit differences in Tie-2 expression, as well [78]. A limited number of CD14^++^CD16^−^ monocytes exhibit Tie-2 expression [90]. A recent genomic analysis confirmed the expression of Tie-2 in the CD14^++^CD16^+^ population [40,91,92]. Tie-2 is expressed primarily, but not entirely, in CD16^+^ monocytes and, to a lesser extent, in CD16^−^ monocytes. This conclusion comes from studies showing that Tie-2 is expressed by intermediate monocytes and non-classical monocytes [9,93]. The non-classical monocyte (CX3CR1^high^/Ly6C^low^) population has the ability to prevent metastasis in a handful of murine cancer models—e.g., PyMT breast cancer, induced LLc or B16-F10 cancer cells injected intravenously [9]. However, human studies show that SLAN^+^ cells are absent from solid cancer, in both primary and metastatic sites [94]. Interestingly, the population of SLAN-positive cells—based on their morphology, lymphoma immature dendritic cells (DCs)—and macrophages were detected in high density in nodal and extranodal locations of numerous types of non-Hodgkin lymphomas, particularly the diffuse large B-cell lymphoma [95].

Another similarity between TEMs and non-classical monocytes is the lack of C-C motif chemokine receptor 2 (CCR2) on their surface. CCR2 is an important receptor for the monocyte chemoattractant protein-1 (MCP1), a chemokine that attracts monocytes to inflamed tissues [35,44,90]. The absence of this receptor is also observed in CD14^low^CD16^+^SLAN^+^ monocytes [94].

The Ang-Tie pathway is well-described in endothelial cells. Limited data are available on this mechanism in TEMs [74]. In humans, the Ang-Tie signalling complex consists of (1) receptors, Tie-1, and, most importantly, Tie-2, and (2) its ligands, angiopoietins (1, 2, and 4) [44]. According to the literature, all these elements are involved in the Tie-Ang pathway’s functioning. However, Ang-1, Ang-2, and Tie-2 play a predominant role here. Under physiological conditions, Ang-1 binds to Tie-2, as a result of which the receptor is autophosphorylated. This leads to the activation of the AKT1/PI3K pathway. Subsequently, FOXO1 is phosphorylated, which means that its activity—modulating the expression of genes involved in blood vessel destabilisation and apoptosis—is inhibited [41,74]. This induces the stabilisation of newly formed vessels [86]. Angiopoietin-2 binding to Tie-2 can result in two different scenarios—the same agonist effect as in Ang-1 described above or the opposite one. The Tie-2 receptor is not active, which means that FOXO1 is not phosphorylated and can serve its role. The end result of Ang-2-mediated Tie-2 activation is vascular destabilisation. The latter scenario happens as a result of TNF (inflammatory cytokine) action—cleaving of the Tie-1 receptor, which, in turn, decreases Ang-2-dependent Tie-2 activation [74] or excessive Ang-2 concentration produced by inflammatory endothelial cells and tumour tissue [86]. In their murine EOC study, Zhang et al. [55] showed that tumour-derived VEGF caused the upregulation of Ang-2 in host stroma endothelial cells. They also found that the upregulation of Ang-2 in these cells was related to the disorganisation of the pericyte layer, cell loss, and vascular instability in the area surrounding the bulk.

At this point, it is worth emphasising the role of VEGF’s presence in Ang-2 activity. These two factors cooperate in the processes of angiogenesis and vessel destabilisation [16]. Studies have shown that, in the presence of high concentrations of VEGF, Ang-2 activates the process of angiogenesis [41]. Conversely, the absence of VEGF results in Ang-2 promoting vascular regression by inducing the apoptosis of endothelial cells [96,97]. As mentioned before, TEMs produce and secrete VEGF [45,51]. What is more, there is research showing that M1 macrophages can also express VEGF receptors (VEGFRs) on their surface [98]. Therefore, one of the key questions in determining the balance of *Ang1*/*Ang2* function may be whether Tie-2-expressing macrophages also express VEGF and VEGFR.

**Figure 1:**
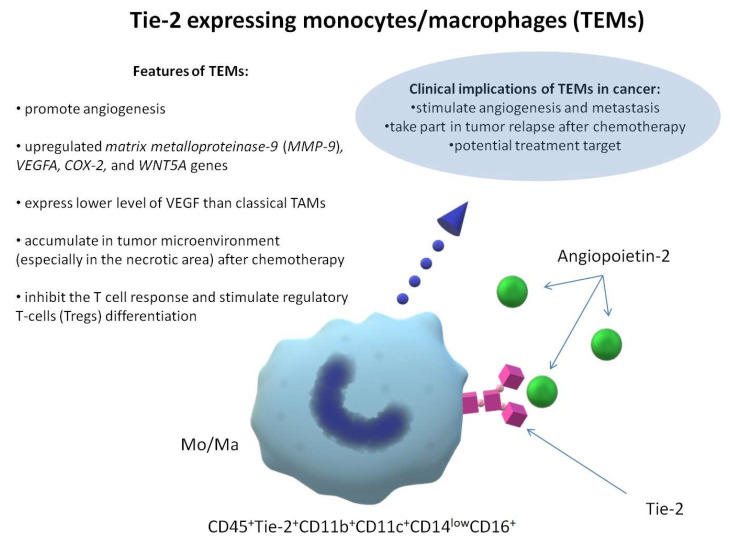
Existing knowledge about Tie-2-expressing monocytes/macrophages.

There are limited data concerning the Ang-Tie pathway mechanism. Four pathways are mentioned in the literature—(1) JAK-STAT [88], (2) AKT1 [69,99] (3) ERK, and (4) p38 mitogen-activated protein kinase (MAPK) [99]. JAK-STAT activation in Tie-2-positive macrophages causes increased production of proinflammatory factors [88]. The data on this subject are limited and remain unclear. Tie-2 expression seems to have an impact on their proinflammatory activity. As previously mentioned, Tie-2 is a receptor that has a proangiogenic effect in the endothelial cells. However, whether Tie-2 has an actual impact on the proangiogenic activity of TEMs remains unclear. Existing knowledge about TEMs is summarised in Figure 1.

## 5. Homing Mechanisms for TEMs

Recruitment of TAMs depends on multiple factors, but TEMs are slightly different. These differences arise from the absence or presence of specific molecules on the TEMs’ surface, as described below.

The homing mechanism for TEMs is cytokine-gradient-dependent. These cells migrate along CCL-3, CCL-5, CCL-8, CCL-12, and most importantly, Ang-2 [11,31].

TEMs express the chemokine receptor CXCR4 and can be attracted into tumours by CXCL12. The anti-CXCR4 antibody blocking of the CXCL12/CXCR4 axis was found to significantly reduce TEMs, but not Tie-2^–^ TAMs, recruitment to combretastatin-A4-phosphate (the vascular-disrupting agent)-treated tumour in a murine model [100].

Ang-2 is a powerful chemoattractant for TEMs. Several studies have shown that TEMs migrate towards higher concentrations of Ang-2 [35,90,101,102]. Wang et al. [35] indicated that Ang-2-concentration-dependent migration of TEMs further resulted in the stimulation of endothelial cells by IGF1 signalling. The impairment of this signalling pathway by blocking IGF1 caused the proangiogenic and prometastatic activity of TEMs to decrease. Surprisingly, Scholz et al. demonstrated that it was not Tie-2-Ang-2-pathway-dependent migration. The key factor was presumably the increased monocyte/macrophage adhesion mediated via β2-integrins [102].

Interestingly, the recently identified protumour macrophage population—CCR-2^+^Tie-2^−^ macrophages—was shown to migrate towards primary tumour and metastatic lesions in an Ang-2-dependent manner. The blockade of Ang-2 resulted in decreased CCR-2^+^Tie-2^−^ monocyte infiltration due to a suppressed proinflammatory pattern of endothelial cells [103].

The absence of L-selectine (CD62L), another molecule responsible for the attraction of monocytes to the inflammatory area, is also characteristic of TEMs [104].

Data on factors attracting TEMs are contradictory and require further research.

## 6. Treatment Targeting the Proangiogenic Pathways and Perspectives

The standard of care for EOC is represented by optimal cytoreductive surgery and cytotoxic platinum-based chemotherapy. Even though the initial response to the carboplatin-paclitaxel treatment is effective, three quarters of patients at FIGO stages III and IV experience cancer recurrences around 15 months after the diagnosis [11]. There is no fully effective treatment for metastatic or relapsed EOC [8]. Attempts to improve the therapeutic efficacy of recurrent EOC chemotherapy by adding hyperthermic intraperitoneal chemotherapy (HIPEC) with carboplatin during secondary cytoreductive surgery did not yield better clinical outcomes [105]. Chemotherapy used as a life-saving therapy may be responsible for creating a population of highly chemo-reluctant tumour cells [17]. The fittest ones, the highly resistant clones that survive chemotherapy treatment, are responsible for cancer recurrence, since they are the only tumour cells left. One promising treatment approach for EOC patients involves immune checkpoint inhibitors, including the programmed cell death pathway (PD-1/PD-L1). Despite the success of PD-1 and PD-L1 inhibitors in other solid tumours, such as melanoma, lung, and renal cancer, the low response rate in OC patients is disappointing and may be related to the density of tumour-infiltrating lymphocytes (TILs). It is well known that the application of PD-1/PD-L1 in cancer patients with “‘hot” tumours, i.e., with a high density of TILs, is beneficial. However, EOC appears as a “cold” or “warm” tumour with low to intermediate levels of TILs, which may hinder the success of immunotherapy [106,107]. The recent clinical trials focused on the PD-1/PD-L1 pathway in OC patients have established that the overall response rates are in the range of 11‒24%. However, many factors involved in the effectiveness of this therapy are still unknown [108].

The recent discoveries in cancer molecular biology and its mechanisms and alternations have made a personalised therapeutic approach for EOC more viable [2,8]. Standard therapeutic options for EOCs have been expanded to include bevacizumab—a VEGF inhibitor (VEGFi), a selectively VEGF-binding monoclonal antibody [8]—as well as olaparib, niraparib, and rucaparib, and poly ADP-ribose polymerase (PARP, an enzyme involved in DNA repair) inhibitors (PARPi) [109]. 

Data provided by clinical trials on bevacizumab [110,111] and PARPi [112,113,114,115,116,117] prove that these treatment methods are effective [109]. Despite their undisputed advantages, regrettably, their usage is associated with adverse side effects and considerable costs [64]. 

In order to investigate the effectiveness of immuno-antiangiogenic chemotherapy combinations, as well as the implications of the sequence of therapeutic intervention (a surgery followed by drugs, or a reverse sequence), Moore et al. [118] analysed data on over 1200 EOC patients. They found that there were no significant differences in overall survival (OS) and progression-free survival (PFS) rates between groups treated with atezolizumab, bevacizumab, and chemotherapy, proving that PD-L1-targeting immunotherapy was ineffective in newly diagnosed EOC.

In their murine EOC model studies, Pulaski et al. [52] provided data about the efficacy of alemtuzumab as a targeted drug against myeloid-derived cells, named by them as vascular leukocytes, including TEMs. However, the target was not connected with the Tie-Ang mechanism. Alemtuzumab is the anti-CD52 drug, since CD52 happens to be expressed on vascular leukocytes. Another study on EOC was performed by Monk et al. [119]. In the TRINOVA-1 randomised double-blind phase 3 study, they provided data about trebananib (AMG 386; Amgen, Thousand Oaks, CA, USA) and its effectiveness in recurrent EOC patients. The mechanism of this drug is based on blocking ligands for Tie-2, Ang-1, and Ang-2. They found that the inhibition of the Tie-2-Ang pathway with trebananib provided a clinically significant extension of PFS. Another phase 3 study, ENGOT-ov-6/TRINOVA-2 (NCT01281254), also focused on trebananib. In this study, the trebananib-pegylated liposomal doxorubicin combination showed no improvement of PFS in recurrent EOC. However, the solution proved to have anticancer effects as indicated by the improved objective response rate and duration of response [120,121]. To evaluate the effectiveness of conventional EOC chemotherapy in combination with bevacizumab, in ICON7 [63], the plasma concentration of soluble Tie-2 (sTie-2) was investigated. The study demonstrated that sTie-2 coupled with Ca-125, an unspecific EOC biomarker, provided information on the vascular response and the vascular progression of EOC in VEGFi-treated OC patients [63]. Later studies showed that sTie-2 concentration in plasma was a vascular response biomarker useful in the optimisation of VEGFi application in EOC patients [64]. According to ClinicalTials.gov, a pilot clinical study (NCT04770376) is being conducted to evaluate soluble forms of Tie-2, Ang-1, and VEGF as predictive markers for women with advanced OC treated with bevacizumab. What is more, the clinical study VALTIVE 1 (NCT04523116) is currently recruiting OC patients at FIGO stage IIIC or IV to evaluate the role of Tie-2 as a response biomarker for patients treated with VEGFi, i.e., bevacizumab. However, no findings have been published yet [122,123].

Targeting TEMs by the Ang-Tie-2 kinase signalling pathway was the aim of the study conducted by Harney et al. [124] on a murine model of metastatic mammary carcinoma and pancreatic neuroendocrine tumours. This work emphasises the potential of rebastinib in therapies for solid carcinomas, which are characterised by the TME being significantly infiltrated by Tie-2-expressing macrophages.

Scientific reports on the features of microvessels and their potential use in evaluating the prognosis and aggressiveness of EOC [125] and the effectiveness of antiangiogenic drugs [110,111] and plasma Tie-2 concentration application as a biomarker for antiangiogenic therapy [64] highlight the importance of research on EOC vasculature.

## 7. Conclusions

Throughout the years, the body of knowledge about EOC has been significantly expanded. Much more is known about the sites of origin, molecular alternations, and the biology of EOC, as well as about the tumour niche. However, detection and treatment methods are still not effective enough. Therefore, it seems important to search for immunological triggers in patients with different clinical manifestations of the disease. 

Angiogenesis is a critical process not only for the progression, but also for the existence of tumours. Its stimulation is probably the key role of TEMs. However, their contribution to OC angiogenesis is ambiguous and remains to be further investigated. Some studies have confirmed the role of TEMs in tumour progression and metastasis. TEMs have been implicated in suppressing T-cell proliferation and weakening antitumoural T-cell immune response. However, the molecular background of the proangiogenic and immunosuppressive activity of TEMs in EOC is still not fully explained. Moreover, it is currently unclear whether TEMs’ infiltration, phenotype, and activity are associated with the clinical characteristics of EOC—e.g., the FIGO stages, the Kurman and Shih types of tumour, the histological differentiation (grade), menopause status, or the level of biomarkers, e.g., Ca-125, HE4. It is unknown if TEMs’ presence and activity differ between cold and hot ovarian tumours and whether such a finding could be of any clinical and prognostic significance in EOC. Therefore, it is purposeful to search for the immunological causes of the differences in the dynamic and complex immunosuppressive TMEs of human OC and their influence on the clinical manifestation of the disease, treatment, and patient prognosis. Further pre- and clinical trials are clearly necessary to develop biomarkers to select the right group of EOC patients for whom antiangiogenic treatment will be beneficial and to monitor the therapeutic response in real-time. What is more, soluble forms of Tie-2 or Ang-2 may be potential predictors of EOC patients’ response to this type of treatment. The identification of these predictors in patients’ blood is minimally invasive. Hence, liquid biopsy is a useful tool in the prediction of EOC patients’ outcomes.

Understanding the role of TEMs during cancer progression is a prerequisite for devising therapeutic strategies to tackle the tumour at a specific evolutionary stage and select the most suitable therapy.

## Figures and Tables

**Table 1:** Expression of Ang-2 and Tie-2 in ovarian cancer.

Investigated Factor		*N*	Results/Clinical Significance/Treatment Efficiency
**Angiopoietin-2**	**Peripheral blood/plasma/serum**	150 patients: 41 benign tumours, 14 borderline tumours, 95 carcinomas, 10 metastases in the ovary, 19 endometrial carcinomas; 34 women with healthy ovaries	Serum levels of Ang-2 higher in OC patients than in patients with benign or borderline tumours. A relationship between higher serum levels of Ang-2 and primary residual tumour greater than 1 cm after debulking surgery. A positive correlation between higher serum levels of Ang-2 and an advanced tumour stage, poor progression-free survival (PFS) and overall survival (OS) [57].
		75 patients: 38 benign tumours, 18 borderline tumours, 75 carcinomas; 31 women with healthy ovaries	An elevated concentration of angiogenesis-related growth factors in OC patients’ blood. A relationship between the serum level of Ang-2 and poor OS. A relationship between the Ang-2/VEGF receptor 2 (VEGFR-2) ratio and short recurrence-free survival (RFS) [58].
		37 samples (17 EOC and 20 benign cysts)	Serum levels of Ang-2 in EOC patients significantly higher than in patients with benign cysts [35].
	**Ascites**	69 samples	No statistically significant differences in ascites’ sAng-2 levels between sensitive and resistant groups in the protein array [62].
		14 samples	Ang-2 levels significantly higher in ascites than in EOC patient-matched serum [35].
	**Tumour tissue**	52 samples	A statistically significant correlation between the level of VEGF and Ang-2 mRNA expression in ovarian cancer tissue [55].
		85 samples	A relationship between a lower Ang-1/Ang-2 ratio and poor OS (univariate analysis) [54].
		21 patients	No correlation between the expression of Ang-2 and grade, stage, histopathological types, ascites formation, microvessel density, and patient age [59].
**Tie-2/Ang-2**	**Peripheral blood/** **plasma/serum**	91 patients	A higher Ang-1/Tie-2 ratio improved PFS in bevacizumab-treated patients [60].
		92 patients	Tie-2 constitutes a vascular progression marker for OC patients treated with bevacizumab. A combination of Tie-2 and Ca125 provides a significant improvement in the prediction of progressive disease in patients receiving bevacizumab in comparison with Ca125 alone [63].
		92 patients	sTie-2 concentration in plasma constitutes a vascular response biomarker useful in the optimisation of VEGF inhibitor (VEGFi) usage in EOC patients [64].
	**Tumour tissue**	19 patients	No difference in *ANG2* and *TIE2* expression in cancer vs. benign tumours [53].
		12 samples	Expression levels of Ang-2 and Tie-2 higher in OC than in benign ovarian lesions. Upregulated Ang-2 and Tie-2 levels in the early stage of OC compared with matched benign lesions. No difference between early-stage and late-stage OC [56].
		12 primary serous OC, 12 omental metastatic serous OC samples, 10 normal ovaries	Higher expression of Ang-2 in human serous ovarian cancer (including primary and secondary cancer) vs. normal ovaries [61].
		86 patients (86 primary high-grade EOC, 16 omental metastases)	Higher expression of Tie-2 and Ang-2 in metastatic lesions. A relationship between a higher expression of Tie-2 and a low expression of Ang-2 and OS [8].
		32 patients: (22 advanced-stage of OC, 10 benign gynaecological diseases)	Reduction of *ANG1* expression in tumour tissue. A higher *ANG2/1* ratio in the EOC tumour-free peritoneal tissue compared to the control. A decreased expression of *ANG1* and *TIE2* in EOC patients’ peripheral muscle samples compared to non-cancer control tissue. The possible contribution of a high *ANG2*/*ANG1* ratio to systemic vascular leakage and hemodynamic deterioration during tumour debulking surgery [65].
**TEMs**	**Peripheral blood/** **plasma/serum** **and ascites**	114 samples: (PB from 30 EOC, 52 benign ovarian cysts, 20 healthy female donors, and ascites from 7 EOC patients)	A significantly higher percentage of TEMs in peripheral blood and ascites in EOC patients [35].
	**Tumour** **tissue**	12 samples (10 EOC and 2 normal ovaries)	Expression of the Tie-2 on the majority of vascular leukocytes [52].
		Tissue slides from 124 EOC patients and 75 patients with benign ovarian cysts	A significantly higher percentage of TEMs in malignant foci in EOC patients. A positive relationship between the proportion of TEM tumour microvascular density [35].

## Data Availability

All data generated or analysed in this study are included in this publication.
